# PHD3-VHL axis controls HIV-2 infection through oxygen-dependent hydroxylation and degradation of Vpx

**DOI:** 10.1371/journal.ppat.1013241

**Published:** 2025-06-16

**Authors:** Kei Miyakawa, Kiho Tanaka, Yoko Ino, Yayoi Kimura, Taichi Kameya, Fuminori Mizukoshi, Mayuko Nishi, Masaru Yokoyama, Jun Nakabayashi, Masako Nomaguchi, Hironori Sato, Hirokazu Kimura, Hirofumi Akari, Tomoyuki Miura, Akinori Takaoka, Hideki Hasegawa, Tetsuro Matano, Yoji Andrew Minamishima, Akihide Ryo

**Affiliations:** 1 AIDS Research Center, National Institute of Infectious Diseases, Japan Institute for Health Security, Tokyo, Japan; 2 Influenza Research Center, National Institute of Infectious Diseases, Japan Institute for Health Security, Tokyo, Japan; 3 Department of Microbiology, Yokohama City University School of Medicine, Yokohama, Japan; 4 Advanced Medical Research Center, Yokohama City University, Yokohama, Japan; 5 Department of Bioinformatics and Integrative Omics, National Institute of Infectious Diseases, Japan Institute for Health Security, Tokyo, Japan; 6 Life Science Laboratory, Technology and Development Division, Kanto Chemical Co., Inc., Isehara, Japan; 7 Institute of Liberal Arts, Institute of Science Tokyo, Ichikawa, Japan; 8 Department of Microbiology, Tokushima University Graduate School of Biomedical Sciences, Tokushima, Japan; 9 Department of Health Science, Graduate School of Health Sciences, Gunma Paz University, Takasaki, Japan; 10 Center for the Evolutionary Origins of Human Behavior, Kyoto University, Inuyama, Japan; 11 Institute for Life and Medical Sciences, Kyoto University, Kyoto, Japan; 12 Division of Signaling in Cancer and Immunology, Institute for Genetic Medicine, Hokkaido University, Sapporo, Japan; 13 Institute for Vaccine Research and Development, Hokkaido University, Sapporo, Japan; 14 Institute of Medical Science, University of Tokyo, Tokyo, Japan; 15 Department of Biochemistry, Gunma University Graduate School of Medicine, Maebashi, Japan; Institut Cochin, INSERM U1016, FRANCE

## Abstract

HIV-2 viral protein X (Vpx) plays a pivotal role in antagonizing the host restriction factors, including SAMHD1 and components of the HUSH complex, to facilitate viral replication. However, the regulatory mechanisms controlling Vpx stability remain unclear. In this study, we identify the von Hippel–Lindau (VHL) tumor suppressor as a novel E3 ubiquitin ligase that specifically targets Vpx for proteasomal degradation. Mechanistically, we demonstrate that VHL-mediated degradation depends on the oxygen-dependent hydroxylation of Vpx at proline residue 41 (Pro41), a modification catalyzed by prolyl hydroxylase domain-containing protein 3 (PHD3). Using an integrated approach combining crosslinking mass spectrometry and molecular modeling analyses, we elucidate the structural architecture of the PHD3-Vpx complex, revealing the spatial orientation of the catalytic domain of PHD3 required for Pro41 hydroxylation. Furthermore, we establish the physiological significance of this pathway in human macrophages, where pharmacological inhibition or genetic ablation of VHL or PHD3 enhances HIV-2 infection by facilitating Vpx-mediated SAMHD1 degradation. Collectively, our findings unveil a previously unrecognized oxygen-sensitive regulatory mechanism influencing HIV-2 infection and suggest novel therapeutic strategies targeting Vpx stability through modulation of its prolyl hydroxylation status.

## Author summary

Our study reveals a previously unknown mechanism for how human cells control HIV-2 infection. We discovered that a cellular protein called VHL, known for its role in preventing cancer, also helps protect cells against HIV-2 by controlling the levels of a viral protein called Vpx. HIV-2 uses Vpx to overcome our body’s natural antiviral defenses, but we found that cells can fight back by marking Vpx for destruction through a process that requires oxygen. This process involves two key players: an enzyme called PHD3 that attaches oxygen-dependent modifications to Vpx, and VHL that recognizes these modifications and targets Vpx for disposal. This is particularly interesting because it shows how cells can use oxygen-sensing machinery, typically involved in helping cells adapt to low oxygen conditions, as a defense mechanism against viruses. By understanding how cells naturally combat HIV-2, we may be able to develop new therapeutic strategies that enhance these defenses.

## Introduction

The molecular interplay between viral and host proteins fundamentally influences the outcome of viral infections and pathogenesis. Human immunodeficiency viruses (HIVs) encode various accessory proteins that facilitate infection, and their interactions with host proteins have been extensively studied. HIV-2, although less prevalent globally than HIV-1 [1–3], exhibits distinct clinical features, including slower disease progression and reduced transmission rates, suggesting distinct virus-host interactions compared to HIV-1.

Among the distinguishing features of HIV-2, viral protein X (Vpx) contributes to its uniqueness through its incorporation into virions. A primary function of Vpx is to counteract the host restriction factor sterile alpha motif and HD domain-containing protein 1 (SAMHD1) by targeting it for proteasomal degradation [4–6]. In nondividing cells such as macrophages, SAMHD1 limits viral infectivity by reducing cellular dNTP concentrations required for efficient reverse transcription. The Vpx-mediated targeting occurs through the recruitment of the Cullin-RING ubiquitin ligase (CRL) complex and the E3 ligase substrate receptor DCAF1, which enables successful viral genome integration in otherwise restrictive myeloid cells [7]. The absence of Vpx in HIV-1 partially explains the differences in cellular tropism between HIV-1 and HIV-2, particularly in myeloid cells.

Despite its small size (approximately 16 kDa), Vpx engages in multiple protein–protein interactions that modulate its function. Beyond SAMHD1 antagonism, recent studies have revealed that Vpx interacts with additional host factors, STING [8], NF-κB [9], and the HUSH complex [10,11], underscoring its multifunctional roles in viral pathogenesis. Specifically, Vpx binds to the HUSH complex components (TASOR, MPP8, and periphilin) and targets them for proteasomal degradation through recruitment of DCAF1 [10]. By counteracting HUSH-mediated epigenetic silencing of viral genes, Vpx promotes HIV-2 gene expression. Notably, Vpx is rarely detected in infected myeloid cells, suggesting the presence of cellular mechanisms that mediate its rapid degradation. However, the identity of these antiviral factors and the biological conditions that trigger their activation remain unknown.

Post-translational modifications play crucial roles in regulating viral protein function and stability [12]. In this regard, we previously demonstrated that PIM kinase-mediated phosphorylation of Vpx enhances its binding affinity for SAMHD1 [13]. Additionally, several studies have demonstrated that Vpx undergoes polyubiquitin modification in infected cells [14]. However, the precise mechanisms controlling Vpx degradation and their implications for HIV-2 pathogenesis remain unclear.

In this study, we performed a screen for E3 ligases that interact with Vpx and identified the tumor suppressor von Hippel–Lindau protein (VHL) as a key regulator of Vpx stability. VHL serves as the substrate recognition component of the CRL2 ubiquitin ligase complex and mediates oxygen-dependent degradation of Vpx through recognition of specific hydroxyproline residues generated by prolyl hydroxylase domain-containing protein 3 (PHD3) under normoxic conditions. Together, these findings demonstrate that PHD3 and VHL function as novel host antiviral factors that control HIV-2 infection by regulating Vpx stability.

## Results

### Identification of VHL as a novel E3 ubiquitin ligase for Vpx

**Fig 1:**
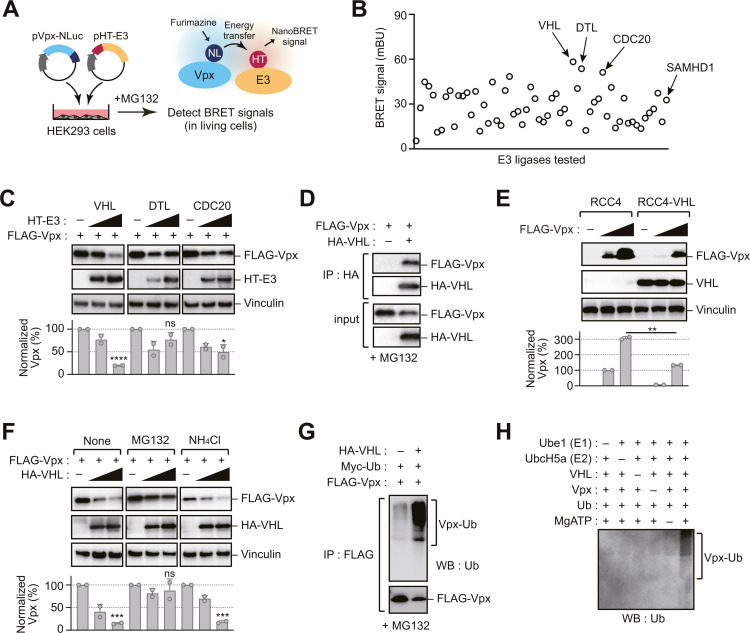
Identification of VHL as a novel E3 ubiquitin ligase for Vpx. (A, B) NanoLuc bioluminescence resonance energy transfer (NanoBRET)-based screen for the identification of Vpx-interacting E3 ligases (58 genes) in living human embryonic kidney 293 (HEK293) cells. Cells were co-transfected with NanoLuc (NL)-conjugated Vpx and HaloTag (HT)-fused E3 ubiquitin ligase expression vectors (A). Following this, HT-618 ligand and furimazine substrate were added in the presence of MG132. Among the tested ligases, VHL showed the highest BRET signal, indicating its strong interaction with Vpx (B). Complete data for all 58 E3 ligases tested are provided in S1 Data. (C) VHL reduces Vpx protein levels. Immunoblotting of HEK293 cells expressing FLAG-Vpx and the indicated E3 ligase (VHL, DTL, or CDC20) revealed a reduction in Vpx levels specifically in cells expressing VHL. (D) Vpx associates with VHL. Immunoprecipitation of HEK293 cells expressing FLAG-Vpx and HA-VHL was performed using an anti-HA antibody. (E) VHL expression decreases Vpx levels. Immunoblotting of FLAG-Vpx in VHL-null RCC4 (RCC4) and VHL-expressing RCC4 (RCC4-VHL) cells demonstrated VHL-dependent Vpx degradation. (F) VHL-induced degradation of Vpx is proteasome-dependent. HEK293 cells transfected with vectors encoding FLAG-Vpx and HA-VHL were treated with MG132 (10 µM) to inhibit proteasomal activity or NH_4_Cl (2 mM) to inhibit lysosomal activity, 16 h before harvest. (G) Ubiquitination of Vpx is increased by VHL. HEK293 cells co-expressing FLAG-Vpx and HA-VHL were immunoprecipitated using an anti-FLAG antibody. The immunoprecipitates were analyzed by immunoblotting with anti-ubiquitin (Ub) and anti-FLAG antibodies. (H) Vpx ubiquitination in an in vitro ubiquitination assay. The indicated components were mixed in the presence or absence of MgATP. Samples were subject to SDS-PAGE, and the gel was processed for immunoblotting with an anti-Ub antibody. Bar graphs in (C, E, F) indicate the ratio of Vpx levels normalized to vinculin, as determined by densitometry. Data are presented as mean ± standard deviation (SD) (n = 2). Statistical significance was evaluated using a two-tailed unpaired *t*-test. ****P < 0.0001; ***P < 0.001; **P < 0.01; *P < 0.05; ns, not significant. Immunoblots are representative of experiments with similar results (n ≥ 2).

Previous studies have demonstrated that cellular Vpx protein levels are regulated via ubiquitin-dependent proteasomal degradation [14]. To identify the specific E3 ubiquitin ligase responsible for this regulation, we conducted a screening system utilizing NanoLuc bioluminescence resonance energy transfer (NanoBRET) technology (Fig 1A). This proximity-based assay enables the detection of protein-protein interactions in living cells with high sensitivity and low background signal. We generated a NanoLuc-tagged Vpx (NL-Vpx) fusion protein as the energy donor and screened it against a library of 58 HaloTag-fused E3 ubiquitin ligases (HT-E3s) as potential energy acceptors. When co-expressed in HEK293 cells, protein-protein interactions bring the NanoLuc and HaloTag moieties into close proximity, resulting in energy transfer that can be quantitatively measured as a BRET signal. This screen identified the tumor suppressor protein VHL as a potential Vpx interactor, alongside DTL and CDC20 as secondary candidates with high BRET signals (Fig 1B). Co-expression studies revealed that VHL, but not DTL or CDC20, reduced Vpx protein levels in a dose-dependent manner (Fig 1C). The interaction between VHL and Vpx was validated through co-immunoprecipitation assays (Fig 1D). Consistent with these findings, Vpx expression levels were significantly higher in VHL-null carcinoma cell line, RCC4 cells, than in VHL-expressing RCC4 cells (Fig 1E). The VHL-mediated reduction in Vpx levels was blocked by treatment with the proteasome inhibitor MG132 but not the lysosome inhibitor NH_4_Cl (Fig 1F), confirming a proteasome-dependent pathway. To characterize this pathway, we performed ubiquitination assays and found that Vpx underwent extensive polyubiquitination in the presence of VHL (Fig 1G). Furthermore, in vitro ubiquitination assays with purified E1 and E2 proteins demonstrated that VHL directly mediates Vpx polyubiquitination (Fig 1H). Collectively, these results identify VHL as a novel E3 ligase that interacts with and regulates Vpx stability.

### Molecular requirements for VHL-mediated Vpx degradation

**Fig 2:**
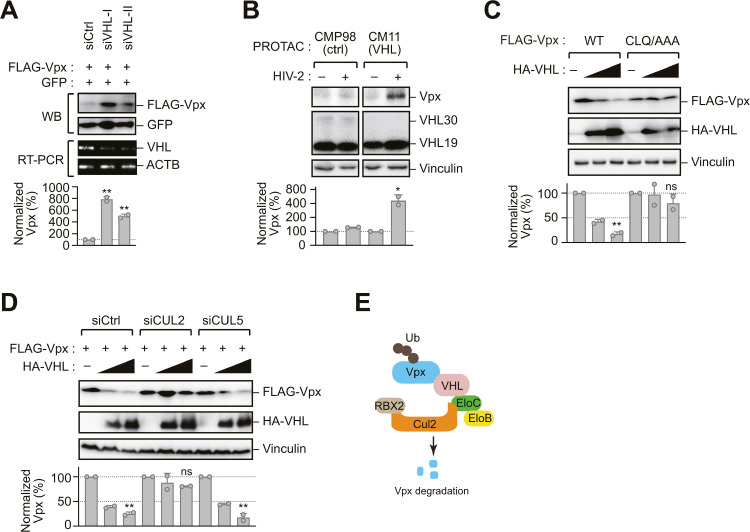
Molecular requirements for VHL-mediated Vpx degradation. (A) VHL depletion enhances Vpx expression. HepG2 cells, which express high levels of endogenous VHL, were treated with two siRNAs targeting VHL (siVHL-I and siVHL-II) or a non-targeting control siRNA (siCtrl). FLAG-Vpx and GFP (control) were then expressed, followed by immunoblotting to assess protein levels and RT-PCR analysis to confirm VHL knockdown. (B) VHL-targeting proteolysis-targeting chimera (PROTAC) enhances Vpx expression. PMA-differentiated Mono Mac 6 cells were treated with CMP98 (control PROTAC) or CM11 (pVHL30-targeted PROTAC) and then infected with HIV-2 for 2 d. Immunoblotting of cells with anti-Vpx and anti-VHL antibodies showed that CM11 inhibits VHL-mediated degradation of Vpx. (C) A VHL mutant lacking the EloB/C-binding motif does not affect Vpx expression. Immunoblotting of HEK293 cells expressing FLAG-Vpx and HT-VHL (WT or CLQ/AAA). (D) CUL2 depletion attenuates VHL-induced Vpx degradation. HEK293 cells were transfected with control (Ctrl), CUL2-specific, or CUL5-specific siRNAs and co-expressed FLAG-Vpx and HA-VHL. (E) Predicted E3 ligase complex for Vpx ubiquitination and degradation. A schematic illustration depicts the predicted components, including VHL, EloB/C, CUL2, and Rbx1, involved in the ubiquitination and proteasomal degradation of Vpx. Bar graphs in (A–D) indicate the ratio of Vpx levels normalized to vinculin, as determined by densitometry. Data are presented as mean ± SD (n = 2). Statistical significance was evaluated using a two-tailed unpaired *t*-test. **P < 0.01; *P < 0.05; ns, not significant. Immunoblots are representative of experiments with similar results (n ≥ 2).

To validate the physiological relevance of VHL-mediated Vpx regulation, we examined the effects of manipulating endogenous VHL levels. siRNA-mediated knockdown of VHL resulted in increased Vpx protein levels in HepG2 cells, which express robust levels of endogenous VHL (Fig 2A). Consistent with this finding, treatment with VHL-targeting proteolysis-targeting chimeras (PROTACs) led to elevated Vpx levels in HIV-2-infected PMA-differentiated Mono Mac 6 cells (Fig 2B). VHL functions as the substrate recognition component of the CRL2^VHL^ E3 ubiquitin ligase complex, which comprises Cullin 2 (CUL2) as the scaffold protein and the Elongin B/C (EloB/C) heterodimer as adaptor proteins [15]. To investigate the molecular requirements for VHL-mediated Vpx degradation, we analyzed the contributions of each complex component. A VHL mutant lacking the BC-box motif required for EloB/C binding (CLQ/AAA mutant) failed to promote Vpx degradation (Fig 2C). Similarly, depletion of CUL2, but not CUL5, via RNA interference impaired Vpx degradation (Figs 2D and S1A). Collectively, these findings demonstrate that the CRL2^VHL^ E3 ligase complex is a key regulator of Vpx protein stability (Fig 2E).

### Impact of VHL depletion on HIV-2 infection

**Fig 3:**
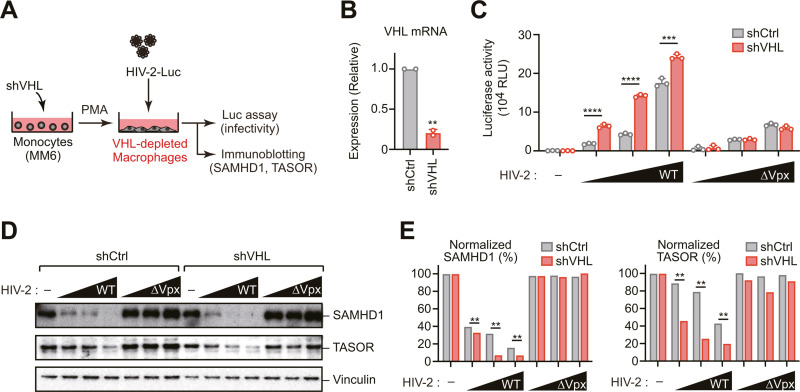
Impact of VHL depletion on HIV-2 infection. (A–E) Schematic representation of the single-cycle HIV-2 infection assay (A). Mono Mac 6 (MM6) cells were stably transduced with shRNA targeting VHL. VHL mRNA levels were quantified by RT-qPCR to confirm knockdown efficiency (B). The cells were then differentiated into macrophage-like cells using phorbol 12-myristate 13-acetate (PMA) treatment and infected with the indicated HIV-2 (WT or ∆Vpx) encoding Luc. Forty-eight hours after infection, HIV-2 infectivity was measured via a luciferase assay (C) and protein levels of SAMHD1 and TASOR were assessed via immunoblotting (D). The ratio of SAMHD1 or TASOR levels normalized to vinculin was determined by densitometry (E). Statistical significance was determined using a two-tailed unpaired *t*-test. ****P < 0.0001; ***P < 0.001; **P < 0.01. Immunoblots are representative of experiments with similar results (n ≥ 2).

To elucidate the biological significance of VHL-mediated Vpx regulation in the context of viral infection, we generated PMA-differentiated Mono Mac 6 cells with shRNA-mediated VHL knockdown and evaluated their susceptibility to HIV-2 infection using a luciferase reporter virus system (HIV-2-Luc) (Fig 3A and 3B), as previously described [13]. This approach enabled quantitative assessment of both viral entry and early post-entry events. VHL-depleted cells exhibited enhanced HIV-2 infectivity in both a dose-dependent and Vpx-dependent manner compared to control cells (Fig 3C). Consistent with increased Vpx stability, SAMHD1 was degraded more efficiently in VHL-knockdown cells following infection with wild-type HIV-2-Luc compared to control cells (Fig 3D and 3E). Similarly, another Vpx substrate, TASOR (a component of the HUSH complex), exhibited enhanced degradation in the absence of VHL (Fig 3D and 3E). Collectively, these results demonstrate that VHL regulates HIV-2 infection by targeting Vpx for degradation, thereby stabilizing antiviral restriction factors such as SAMHD1 and TASOR, which function to limit HIV-2 infectivity.

### Prolyl hydroxylation-dependent VHL-mediated degradation of Vpx

**Fig 4:**
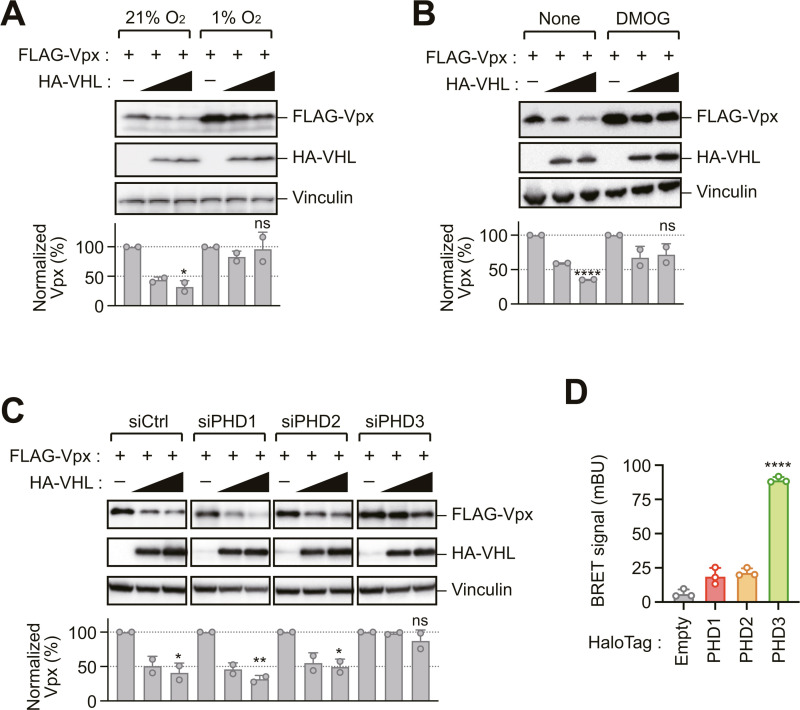
Prolyl hydroxylation-dependent VHL-mediated degradation of Vpx. (A, B) Hypoxic conditions or inhibition of prolyl hydroxylase activity attenuates VHL-induced Vpx degradation. HEK293 cells were transfected with vectors encoding FLAG-Vpx and HA-VHL and cultured under 1% O_2_ conditions (A) or treated with prolyl hydroxylase inhibitor dimethyloxallyl glycine (DMOG) (1 mM) for 16 h before harvest (B). (C) Prolyl hydroxylase domain-containing protein 3 (PHD3) depletion attenuates VHL-induced Vpx degradation. HEK293 cells were treated with either control (Ctrl) siRNA or siRNAs targeting PHD isoforms and transfected with vectors encoding FLAG-Vpx and HA-VHL. (D) NanoBRET analysis of cells expressing NL-Vpx and HT-PHDs demonstrates the specificity of Vpx for PHD3. Bar graphs in (A–C) indicate the ratio of Vpx levels normalized to vinculin, as determined by densitometry. Data are presented as mean ± SD (n = 2). Statistical significance was determined using a two-tailed unpaired *t*-test. ****P < 0.0001; **P < 0.01; *P < 0.05; ns, not significant. Immunoblots are representative of experiments with similar results (n ≥ 2).

The substrate specificity of the VHL E3 ubiquitin ligase complex is primarily determined by recognition of hydroxylated proline residues within target proteins [16,17]. This post-translational modification is catalyzed by members of the PHD-containing family of enzymes, which require molecular oxygen as a co-substrate [18–20]. Therefore, we investigated whether Vpx degradation follows a similar mechanism. Notably, VHL-mediated Vpx degradation was impaired under hypoxic conditions (1% O_2_) (Fig 4A) and upon treatment with the prolyl hydroxylase inhibitor dimethyloxallyl glycine (DMOG) (Fig 4B), indicating that PHD-mediated prolyl hydroxylation of Vpx is a prerequisite for its VHL-dependent degradation. Systematic RNA interference studies targeting individual PHD isoforms revealed that selective depletion of PHD3, but not PHD1 or PHD2, significantly attenuated VHL-mediated Vpx degradation (Figs 4C and S1B). Moreover, NanoBRET analysis demonstrated a preferential binding affinity between PHD3 and Vpx compared to other PHD isoforms (Fig 4D), identifying PHD3-specific hydroxylation as the critical determinant for VHL-mediated Vpx degradation.

### Identification of proline 41 (Pro41) as the PHD3 hydroxylation site in Vpx

**Fig 5:**
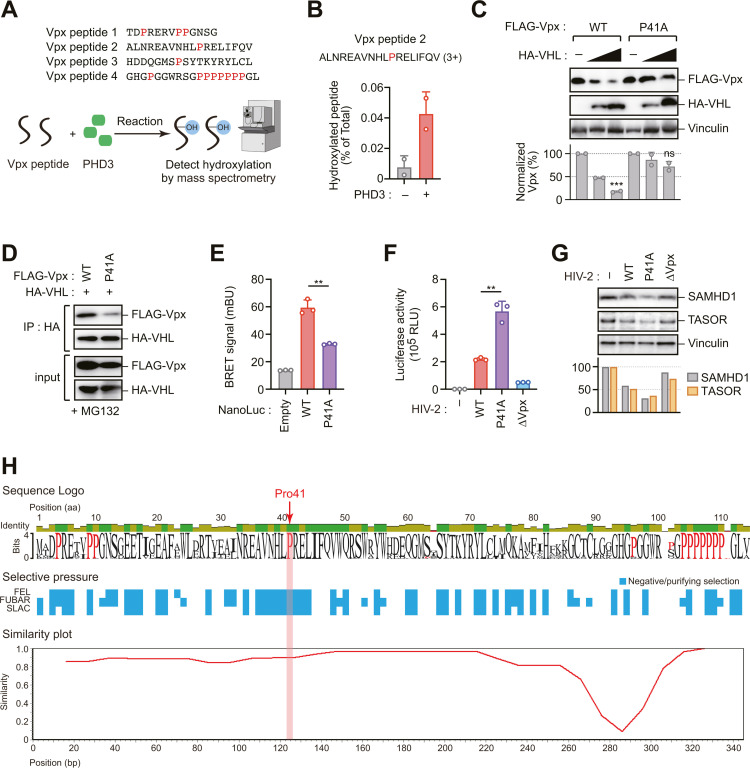
Identification of proline 41 as the PHD3 hydroxylation site in Vpx. (A, B) Hydroxylation of Vpx Pro41 is increased by PHD3. Indicated Vpx peptides were incubated with recombinant PHD3 in hydroxylation reaction buffer and analyzed by mass spectrometry (MS) (A). Hydroxylation rates of Pro41 in Vpx peptide 2 were increased in the presence of PHD3. Mass spectrometry illustration was obtained from TogoTV (https://togotv.dbcls.jp/en/togopic.2025.048.html). (B). (3+) indicates the charge state of the peptide ion detected by mass spectrometry. (C) Mutation of Pro41 to alanine (P41A) renders Vpx resistant to VHL-mediated degradation. Immunoblotting assay of HEK293 cells expressing FLAG-Vpx (WT or P41A) and HT-VHL. Bar graph indicates the ratio of Vpx levels normalized to vinculin, as determined by densitometry. Data are presented as mean ± SD (n = 2). Statistical significance was determined using a two-tailed unpaired *t*-test. ***P < 0.001; ns, not significant. Immunoblots are representative of experiments with similar results (n ≥ 2). (D) Pro41 is required for Vpx-VHL interaction. Co-immunoprecipitation of HEK293 cells expressing HA-VHL and FLAG-Vpx (WT or P41A) was performed using an anti-HA antibody. (E) NanoBRET analysis of HEK293 cells expressing HT-VHL and NL-Vpx (WT or P41A). Data are presented as mean ± SD (n = 3). Statistical significance was determined using a two-tailed unpaired t-test. **P < 0.01. (F, G) HIV-2 containing Vpx-P41A mutant shows enhanced infectivity. PMA-differentiated Mono Mac 6 cells were infected with equal amounts of wild-type HIV-2-Luc or HIV-2-Luc containing the Vpx-P41A mutation. Forty-eight hours after infection, HIV-2 infectivity was measured via a luciferase assay (F) and protein levels of SAMHD1 and TASOR were assessed via immunoblotting (G). The ratio of SAMHD1 or TASOR levels normalized to vinculin was determined by densitometry and shown in bottom panel. Data are presented as mean ± SD (n = 3). Statistical significance was determined using a two-tailed unpaired t-test. **P < 0.01. Immunoblots are representative of experiments with similar results (n ≥ 2). (H) Sequence logo analysis showing amino acid conservation across HIV-2 Vpx sequences (n = 80), with Pro41 highlighted as a highly conserved residue (top panel). Selective pressure analysis revealing negative selection (shown in blue) at the Pro41 region (middle panel). A similarity plot demonstrating high sequence conservation in the N-terminal region containing Pro41, contrasting with greater variation in the C-terminal region (bottom panel).

Having established PHD3 as the key enzyme regulating Vpx stability, we next sought to identify the specific proline residue(s) targeted for hydroxylation. To pinpoint these residues, we employed a biochemical approach utilizing synthetic Vpx peptides corresponding to distinct proline-containing regions (Fig 5A). Mass spectrometry (MS) analysis of in vitro hydroxylation assays with recombinant PHD3 revealed selective modification of peptides encompassing Pro41 (Figs 5B and S2A). In contrast, the hydroxylation status of C-terminal proline-rich sequences remained unaffected by PHD3 activity (S2B Fig). Site-directed mutagenesis studies demonstrated that substitution of Pro41 with alanine (P41A) conferred resistance to VHL-mediated proteolysis (Fig 5C). To determine whether the interaction between Vpx and VHL depends on Pro41 hydroxylation, we performed co-immunoprecipitation and NanoBRET assays. We confirmed that the Vpx P41A mutation reduced the interaction with VHL (Fig 5D and 5E), while it did not affect binding to SAMHD1 or TASOR (S3A and S3B Fig).

Next, we characterized the PHD3-mediated Vpx ubiquitination profile. While some level of Vpx ubiquitination was observed in vitro even in the absence of PHD3 (Fig 1H), supplementation with PHD3 and its cofactors enhanced the ubiquitination profile of Vpx (S3C Fig), suggesting that the in vitro system may support some level of hydroxylation-independent interaction. Importantly, the Vpx P41A mutant exhibited reduced ubiquitination compared to wild-type Vpx even in the presence of PHD3, confirming the critical role of Pro41 in PHD3-mediated ubiquitination (S3D Fig).

To further validate Pro41’s role in regulating HIV-2 infection, we generated HIV-2-Luc reporter viruses containing the Vpx-P41A mutation. This mutant virus exhibited significantly enhanced infectivity compared to wild-type HIV-2 in PMA-differentiated Mono Mac 6 cells (Fig 5F). Immunoblot analysis showed more efficient degradation of SAMHD1 and TASOR with the Vpx-P41A mutant (Fig 5G), suggesting that disrupting the Pro41 hydroxylation site enhances Vpx stability during infection, leading to more effective antagonism of host restriction factors and increased viral infectivity.

Sequence analysis revealed that Vpx is generally conserved across HIV-2 lineages, with Pro41 being identified as a highly conserved amino acid residue (Fig 5H). Pro41 is also highly conserved in the simian immunodeficiency virus sooty mangabey monkeys (SIVsmm) lineage (S4 Fig). Selective pressure analysis indicated that the region surrounding Pro41 is under negative selection, suggesting significant functional constraints against mutations in this region (Fig 5H). Similarity plot analysis demonstrated that although the N-terminal region containing Pro41 displays high homology across HIV-2 variants, evolutionary changes appear to be predominantly concentrated in the C-terminal region (Fig 5H). Collectively, these findings suggest that PHD3 catalyzes the hydroxylation of the highly conserved Pro41 residue in Vpx, rendering it susceptible to VHL-mediated recognition and subsequent degradation.

### Structural characterization of the Vpx-PHD3 molecular interface

**Fig 6:**
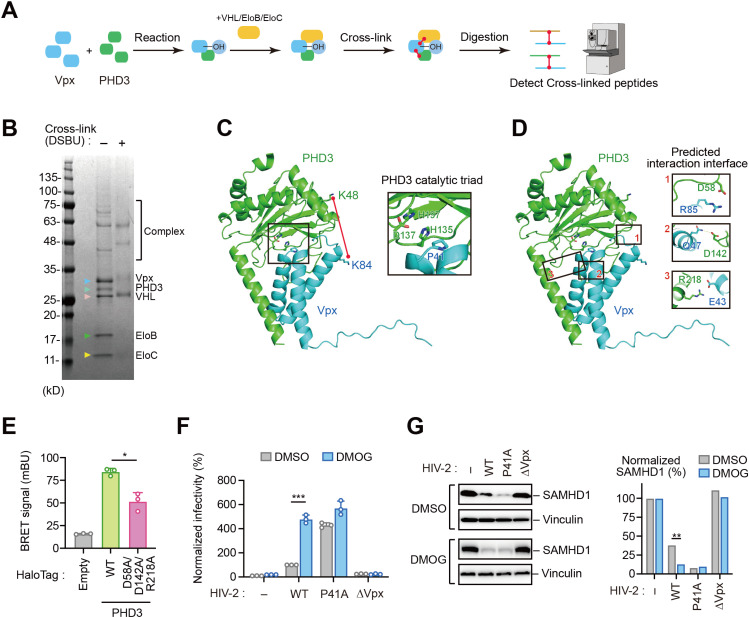
Structural characterization of the Vpx-PHD3 molecular interface. (A) Schematic representation of the experimental procedure of chemical crosslinking MS to analyze the Vpx-PHD3 interaction. Mass spectrometry illustration was obtained from TogoTV (https://togotv.dbcls.jp/en/togopic.2025.048.html). (B) SDS-PAGE gel stained with Coomassie Brilliant Blue to visualize recombinant proteins and their crosslinked products, confirming successful crosslinking. (C, D) Structural insights into the Vpx-PHD3 interface. A docking simulation between Vpx (cyan) and PHD3 (green) shown as cartoon and surface models. Magnified view (right box) of the PHD3 catalytic triad residues highlighted (box) in the complex model (C). The red lines in (C) indicate crosslinked residues detected by MS. Magnified views (right boxes) of three predicted interaction interfaces further elucidate the molecular contacts (D). (E) NanoBRET analysis of HEK293 cells expressing NL-Vpx and HT-PHD3 (WT or D58A/D142A/R218A) demonstrating that PHD3 catalytic site mutations disrupt interaction with Vpx. (F, G) Effect of PHD inhibition on HIV-2 infectivity via a single-cycle HIV-2 infection assay. Primary monocyte-derived macrophages were pre-treated with DMOG and infected with indicated HIV-2-Luc. Luciferase assay (F) and immunoblotting (G) demonstrate reduced viral replication in a Vpx-dependent manner and pronounced SAMHD1 degradation in DMOG-treated cells, respectively. Statistical significance was determined using a two-tailed unpaired *t*-test. ***P < 0.001; **P < 0.01; *P < 0.05. Immunoblots are representative of experiments with similar results (n ≥ 2).

To elucidate the molecular architecture of the Vpx-PHD3 interaction, we employed chemical crosslinking MS utilizing the MS-cleavable crosslinker disuccinimidyl dibutyric urea (DSBU). Recombinant Vpx and PHD3 proteins were incubated in hydroxylation buffer, followed by the addition of VHL and the EloB/C heterodimer to stabilize the complex. The mixture was subjected to DSBU-mediated crosslinking and analyzed by high-resolution liquid chromatography tandem MS (LC-MS/MS) (Fig 6A). SDS-PAGE analysis revealed distinct higher molecular weight species in crosslinked samples compared to non-crosslinked controls, confirming successful intermolecular crosslinking (Fig 6B). MS analysis identified a specific crosslink between Lys85 of HIV-2_Rod10_ Vpx (corresponding to Lys84 in HIV-2_GH-1_ Vpx) and Lys48 of PHD3, delineating a critical interaction interface (S5 Fig). Although intramolecular crosslinks were detected within VHL and Vpx, as well as those expected between EloB and EloC, direct crosslinks between Vpx and VHL were not observable under our experimental conditions.

Based on these experimental data, we generated a molecular model of the Vpx-PHD3 complex. The computational model positions the PHD3 catalytic triad (His135, Asp137, His196) in close proximity to the Vpx Pro41 substrate, with approximately 16 Å separating the crosslinked lysine residues (Fig 6C). Although this distance exceeds the theoretical DSBU spacer length (∼12.5 Å) [21], the discrepancy can be attributed to dynamic lysine side-chain conformations in solution and potential conformational rearrangements during complex formation. To validate this structural model, we engineered a PHD3 variant harboring alanine substitution at residues (Asp58, Asp142, Arg218) predicted to form the Vpx interaction interface (Fig 6D). Quantitative NanoBRET analysis demonstrated that this PHD3 mutant (D58A/D142A/R218A) exhibited significantly reduced binding affinity for Vpx compared to wild-type PHD3 (Fig 6E), providing experimental support for our computational model. Sequence analysis of the putative PHD3-binding interface (Glu43, Gln47, and Arg85) revealed that Glu43 and Gln47 are highly conserved across HIV-2 isolates, while Arg85 exhibits more variability (Fig 5H). This pattern of selective conservation suggests that specific elements of the PHD3-binding interface, particularly Glu43 and Gln47, are under evolutionary constraints that maintain the structural determinants necessary for PHD3 recognition and subsequent hydroxylation of Pro41.

To elucidate the physiological significance of PHD3-mediated regulation in HIV-2 pathogenesis, we evaluated viral infectivity in primary macrophages pre-treated with DMOG. Pharmacological inhibition of prolyl hydroxylation resulted in significantly enhanced wild-type HIV-2 infection, with accelerated and more pronounced SAMHD1 degradation in DMOG-treated primary macrophages (Fig 6F and 6G). Notably, neither the Vpx-P41A mutant nor the Vpx-deficient viruses showed significant changes in infectivity or SAMHD1 degradation following DMOG treatment (Fig 6F and 6G), further corroborating PHD3 as the principal regulatory enzyme controlling Vpx stability and function.

## Discussion

**Fig 7:**
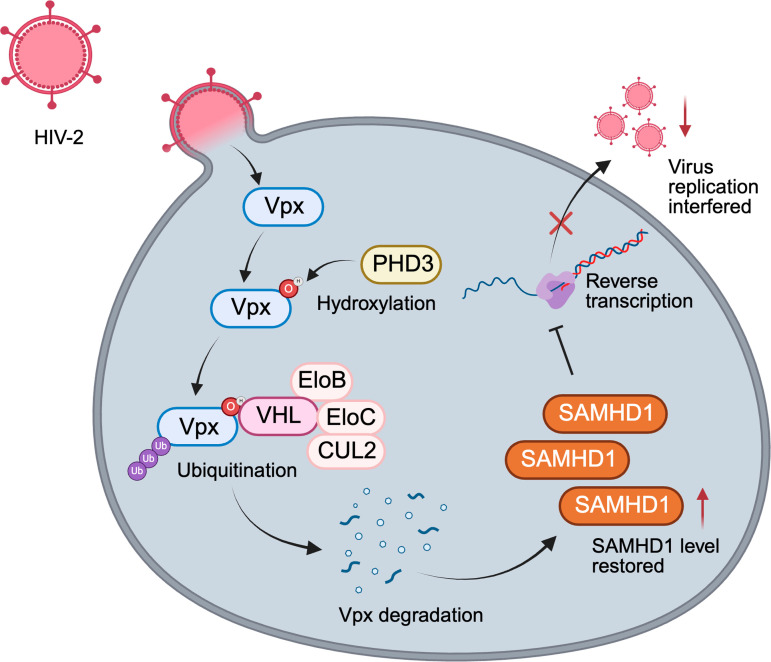
Oxygen-dependent regulation of HIV-2 infection via the PHD3-VHL axis. Schematic model of the oxygen-dependent regulation of HIV-2 infection in macrophages. Upon HIV-2 infection, Vpx undergoes Pro41 hydroxylation catalyzed by PHD3. This modification leads to the recruitment of VHL and formation of the CRL2^VHL^ E3 ligase complex that targets Vpx for proteasomal degradation. This reduction in Vpx levels allows SAMHD1 to maintain its antiviral activity, effectively restricting HIV-2 infection. Created with BioRender.

In this study, we identified a novel regulatory mechanism modulating HIV-2 infection, wherein Vpx undergoes VHL-mediated degradation via the ubiquitin-proteasome system. This mechanism is orchestrated by PHD3-catalyzed, oxygen-dependent prolyl hydroxylation of Vpx (Fig 7). Our findings highlight the critical role of the biological microenvironment in virus-host interactions and provide new insights into viral pathogenesis. This discovery represents a significant advance in our understanding of how cellular oxygen-sensing pathways modulate viral protein stability and function.

We identified a previously uncharacterized post-translational modification of HIV-2 Vpx: hydroxylation by PHD3, followed by VHL-mediated ubiquitination. This cellular defense mechanism releases Vpx-mediated suppression of host antiviral factors SAMHD1 and the HUSH complex, thereby inhibiting early-phase viral replication. Notably, PHD3 enzymatic activity is strictly oxygen-dependent, with hypoxic conditions suppressing Vpx hydroxylation. This oxygen sensitivity has potential implications for viral persistence in relatively hypoxic anatomical reservoirs such as lymph nodes and gut-associated lymphoid tissue [22,23]. In these environments, VHL-mediated Vpx proteolysis is attenuated, potentially facilitating HIV-2 infection. Consistent with this, HIV-2 infection was accelerated when primary macrophages were treated with DMOG. These results suggest a complex interplay between tissue oxygen levels, cellular physiology, and viral protein stability, influencing infection outcomes across different anatomical compartments [24].

Our findings establish Vpx as a novel substrate for the VHL-E3 ubiquitin ligase complex, which includes EloB, EloC, and CUL2. This complex orchestrates the proteasome-mediated proteolysis of target proteins through a highly regulated process [25]. Our in vitro ubiquitination assays revealed that while baseline Vpx ubiquitination can be detected without PHD3, presumably due to the high concentration of reactants, the presence of PHD3 significantly enhances this process, and knockdown of PHD3 reduces Vpx ubiquitination in cellular contexts. Although this ubiquitin-mediated degradation pathway shares features with the canonical hypoxia-inducible factor 1 alpha (HIF1A) degradation pathway, including its requirement for oxygen-dependent prolyl hydroxylation [16,17], the pathway also exhibits several distinct features. Unlike HIF family proteins, which contain the canonical Leu-X-X-Leu-Ala-Pro (LXXLAP) motif required for PHD-mediated hydroxylation [18,20], Vpx lacks this sequence yet undergoes efficient hydroxylation and degradation. Our comprehensive analysis demonstrated that PHD3 could hydroxylate Pro41 in Vpx presumably in an oxygen-dependent manner, adding to growing evidence that the requirements for PHD-mediated hydroxylation are more flexible than previously thought. Recent studies have shown considerable variability in LXXLAP motif recognition [26], supporting the notion that strict sequence consensus is not essential for substrate recognition. However, the structural basis for VHL recognition of hydroxylated Vpx warrants further investigation to comprehensively elucidate this novel protein–protein interaction.

Our molecular characterization of the PHD3-Vpx interaction provided several key insights. Detailed MS analysis identified Pro41 as the specific target for PHD3-mediated hydroxylation, whereas crosslinking MS revealed critical regions (Glu43, Gln47, and Arg85) of proximity between PHD3 and Vpx. Although the detection of hydroxylated peptides posed technical challenges, we successfully identified this modification, directly supporting the proposed regulatory mechanism. The relatively modest levels of hydroxylation observed may reflect the dynamic nature of this modification in vitro or technical limitations in enriching modified species for analysis. Notably, Pro41 exhibits strict conservation across SIVsmm and HIV-2, suggesting strong evolutionary constraints on this residue. Sequence conservation analysis revealed that the N-terminal region containing Pro41, Glu43 and Gln47 is highly conserved, whereas the C-terminal region exhibits greater diversity. This indicates differential selective pressures across distinct domains of the protein, consistent with prior findings that amino acids in the N-terminal region mediate SAMHD1 antagonism [7,27]. This pattern of conservation reinforces the crucial role of the N-terminal region of Vpx, making it a promising target for therapeutic intervention.

The identification of Pro41 as a key regulatory site opens new therapeutic avenues that merit further investigation. Small molecules designed to enhance PHD3-mediated hydroxylation could accelerate Vpx degradation, thereby suppressing viral replication through a novel mechanism. Additionally, VHL-based PROTACs engineered to recognize hydroxylated Vpx represent an innovative strategy for targeted protein degradation that could complement existing antiviral approaches [28]. Investigating the relationship between VHL mutations and HIV-2 susceptibility may illuminate important host genetic factors that influence viral pathogenesis and treatment outcomes, enabling more personalized treatment approaches based on individual genetic profiles and tissue-specific conditions. Furthermore, the oxygen-dependent nature of this regulatory pathway suggests that modulation of tissue oxygen levels or cellular oxygen sensing mechanisms could provide additional therapeutic opportunities.

In conclusion, this study reveals a novel regulatory network through which host factors modulate HIV-2 infection via post-translational modifications and targeted degradation of the viral protein Vpx. These findings not only advance our fundamental understanding of virus-host interactions but also identify new therapeutic strategies for combating HIV-2. Future research should focus on elucidating the structural basis of the VHL-Vpx interaction, developing small molecules targeting this pathway, and exploring tissue-specific effects on viral replication mediated by oxygen-dependent protein degradation. Understanding these mechanisms may lead to novel therapeutic interventions utilizing this regulatory pathway to control HIV-2 infection.

## Materials and methods

### Plasmids

Complementary DNA (cDNA) for Vpx (HIV-2_GH-1_) was synthesized and subcloned into pCMV-FLAG, pcDNA-HA, pCSII-CMV, pEU-E01-His-MCS, or pNLF1-C vectors. Vpx mutants were generated using PCR-based molecular cloning. A plasmid library encoding N-terminal HaloTag-conjugated E3 ubiquitin ligases and PHDs was prepared by Kazusa Genome Technologies (Chiba, Japan) and purchased from Promega. VHL cDNA (Kazusa Genome Technologies, #FHC03983) was subcloned into the pcDNA-HA vector. Plasmids encoding HIV-2-Luc [29] and ubiquitin [30] were utilized as previously described. The HIV-2-Luc reporter virus containing the Vpx-P41A mutation was generated using site-directed mutagenesis.

### Cells

Human embryonic kidney (HEK) 293 (ATCC, #CRL-1573) and HepG2 (ATCC, #HB-8065) cells were maintained in DMEM supplemented with 10% fetal bovine serum (FBS). RCC4 and RCC4-VHL cells were cultured as previously described [31]. Mono Mac 6 cells, a human monocyte-derived cell line exhibiting a mature monocyte phenotype [32], were maintained in RPMI 1640 containing 10% FBS, 1 mM sodium pyruvate, 100 µg/mL insulin, and 1% non-essential amino acids. VHL-depleted Mono Mac 6 cells were generated by transduction with lentiviral particles carrying VHL-specific shRNA (Santa Cruz Biotechnology, #sc-36816-V), followed by selection with 1 µg/mL puromycin. Primary human monocytes (Lifeline Cell Technology, #HC-0002) were differentiated into macrophages by culturing in RPMI 1640 medium containing 10% FBS and 50 ng/mL recombinant human M-CSF (Proteintech, #HZ-1192) for 7 days.

### NanoBRET-based protein–protein interaction assays

NanoBRET assays were performed as previously described [13,33,34]. Briefly, HEK293 cells were seeded into white 96-well plates (2 × 10^4^ cells per well) and transfected with plasmids encoding HaloTag-fused proteins (100 ng) and NanoLuc-fused proteins (1 ng) using Lipofectamine 3000 Transfection Reagent (Thermo) according to the manufacturer’s instructions. Cells were treated with MG132 (10 µM) 16 h prior to NanoBRET measurement. NanoBRET activity was measured 48 h post-transfection using the NanoBRET Nano-Glo Detection System (Promega).

### Vpx degradation assays

HEK293 cells in 12-well plates (2.5 × 10^5^ cells per well) were transfected with plasmids encoding Vpx (100 ng) or VHL (300, or 600 ng) using Lipofectamine 3000 Transfection Reagent (Thermo). In some experiments, cells were treated with MG132 (10 µM) or NH_4_Cl (2 mM) 16 h before harvesting. For siRNA experiments, cells were transfected with siRNA mixtures (20 pmol) 16 h prior to DNA transfection, using HiPerFect Transfection Reagent (Qiagen) or Lipofectamine RNAiMAX Transfection Reagent (Thermo). The siRNAs were obtained from Qiagen and included: CUL2 (#SI02225671, #SI02225678), CUL5 (#SI00052360, #SI00052367), PHD1 (#SI03034738, #SI03109267), PHD2 (#SI04150384, #SI04179196), PHD3 (#SI03023083, #SI03094532), and AllStars Negative Control siRNA (#SI03650318). Protein expression was analyzed by immunoblotting as described below.

### Wheat germ cell-free protein synthesis

Wild-type and P41A mutant HIV-2_GH-1_ Vpx proteins were synthesized using a wheat germ cell-free protein expression system, as previously described [13]. Vpx cDNA was cloned into the pEU-E01-MCS vector (CellFree Sciences) containing a C-terminal 6xHis tag. The plasmids were used as templates for in vitro transcription with SP6 RNA polymerase (CellFree Sciences) to generate mRNAs, which were subsequently used for translation. Cell-free protein synthesis was performed using the WEPRO7240 Expression Kit (CellFree Sciences) according to the manufacturer’s instructions. Briefly, the transcription mixtures containing 1 μg of template plasmid, 80 mM HEPES-KOH (pH 7.8), 16 mM magnesium acetate, 2 mM spermidine, 10 mM DTT, 2.5 mM each of ATP, CTP, GTP, and UTP, 20 units of RNase inhibitor, and 40 units of SP6 RNA polymerase were incubated at 37°C for 3 h. For translation, the transcribed mRNAs were mixed with wheat germ extract (WEPRO7240, CellFree Sciences) and added to the translation buffer (30 mM HEPES-KOH pH 7.8, 100 mM potassium acetate, 2.7 mM magnesium acetate, 0.4 mM spermidine, 2.5 mM DTT, 0.3 mM amino acid mix, 1.2 mM ATP, 0.25 mM GTP, and 16 mM creatine phosphate) in a bilayer format. After incubation at 15°C for 20 h, the synthesized proteins were purified using Ni-NTA agarose (Qiagen) and eluted with 250 mM imidazole. The purity and concentration of the purified proteins were confirmed by SDS-PAGE followed by Coomassie Brilliant Blue staining and compared against BSA standards.

### Immunoprecipitation and ubiquitination assays

Co-immunoprecipitation assays were performed as previously described [13,33,34]. Briefly, HEK293 cells expressing indicated proteins were lysed 48 h post-transfection in lysis buffer (50 mM Tris-HCl pH 7.5, 150 mM NaCl, 1% NP-40, 0.5% sodium deoxycholate, and protease inhibitor cocktail (Roche)). Cell lysates were immunoprecipitated with an anti-HA or anti-FLAG EZview Red Affinity Gel (Merck) for 4–16 h at 4°C with rotation. After washing four times with wash buffer (50 mM Tris-HCl pH 7.5, 150 mM NaCl, 0.1% NP-40), bound proteins were eluted with SDS loading buffer by heating at 95°C for 5 min, and analyzed via immunoblotting. For ubiquitination assays in cells, HEK293 cells were co-transfected with plasmids encoding FLAG-Vpx (WT or P41A), and Myc-ubiquitin. Cells were treated with 10 µM MG132 for 6 h before harvesting. After immunoprecipitation with anti-FLAG EZview Red Affinity Gel (Merck), samples were analyzed by immunoblotting with anti-ubiquitin antibody to detect ubiquitinated Vpx.

### Immunoblotting

Protein concentration was determined using the BCA Protein Assay Kit (Pierce), and approximately 30 μg of total protein per sample was loaded. Samples prepared in SDS loading buffer were separated on 10–20% polyacrylamide gels (Wako Chemicals), transferred onto PVDF membranes (Merck). Membranes were blocked with 5% non-fat dry milk in TBS-T for 1 hour at room temperature before incubation with primary antibodies and probed with primary and horseradish peroxidase (HRP)-conjugated secondary antibodies. Probed proteins were visualized using FluorChem (ProteinSimple) or ImageQuant 800 (Cytiva) imaging systems. Band quantification was performed using ImageJ software (NIH). The following primary antibodies were used: anti-FLAG (Merck, #F3165, 1:2000), anti-HA (MBL Life Science, #M180-3, 1:1000), anti-HaloTag (Promega, #G921A, 1:1000), anti-ubiquitin (Santa Cruz, #sc-8017, 1:500), anti-vinculin (Merck, #V9264, 1:5000), anti-VHL (Cell Signaling, #68547, 1:1000), anti-Vpx (BEI Resources, #ARP-2609, 1:500), anti-TASOR/FAM208A (Merck, #HPA006735, 1:1000), and anti-SAMHD1 (Merck, #SAB1400478, 1:1000). HRP-conjugated secondary antibodies (anti-mouse and anti-rabbit, Cell Signaling) were used at 1:5000 dilution.

### In vitro ubiquitination assays

In vitro ubiquitination reactions (10 µL) contained 1 µg recombinant HIV-2_GH-1_ Vpx or P41A mutant (synthesized using wheat germ cell-free protein synthesis system), 100 nM ubiquitin-activating enzyme E1 (Merck, #SRP6147), 1 µM UbcH5a (R&D Systems, #E2-616), 100 nM EloB/EloC/VHL/CUL2/RBX1 (R&D Systems, #E3-655–025), 5 µM ubiquitin (Merck, #U5507), 2.5 mM MgATP solution (R&D Systems, #B-20), and 1x E3 Ligase Reaction buffer (R&D Systems, #B-71). For PHD3-dependent ubiquitination assays, Vpx was pre-incubated with 300 nM recombinant PHD3/EGLN3 protein (Active Motif, #81033) in PHD3 reaction buffer (20 mM Tris-HCl pH 7.5, 5 mM KCl, 1.5 mM MgCl_2_, 1 mM DTT, 100 µM 2-oxoglutarate, 100 µM ascorbate, and 50 µM (NH_4_)_2_Fe(SO_4_)_2_·6H_2_O) at 30°C for 60 min. Samples were then incubated at 30°C for 60 min and analyzed via immunoblotting with anti-ubiquitin antibody (Santa Cruz, #sc-8017).

### HIV-2 reporter virus production

HIV-2_GH-1_-Luc reporter viruses were prepared by transient transfection of molecular clones into HEK293 cells using a previously established protocol [13]. Briefly, virus stocks were generated by co-transfection of the cells with molecular clones pGL-AN∆Env-Luc (for HIV-2-Luc), pGL-ST∆Env-Luc (for HIV2∆Vpx-Luc) [29] or pGL-AN∆Env-Luc containing the P41A mutation in Vpx (for HIV-2-Luc-VpxP41A) and the VSV-G expression vector at a molar ratio of 3:1. Virus-containing culture supernatants were collected 48 h post-transfection and filtered through a 0.45 µm Millex-HV filter (Merck). For experiments using PROTACs, PMA-differentiated Mono Mac 6 cells were treated with 1 µM of CMP98 (Tocris Bioscience, #6417) or CM11 (Tocris Bioscience, #6416) and infected with HIV-2-Luc. Two days post-infection, cells were subjected to immunoblotting.

### Single-cycle HIV-2 infection assays

Mono Mac 6 cells were seeded in 24-well plates (1 × 10^5^ cells per well). A day before infection, cells were differentiated by treatment with 100 ng/mL phorbol 12-myristate 13-acetate (PMA). Alternatively, monocyte-derived macrophages were seeded in 24-well plates (2 × 10^5^ cells per well). Cells were then infected with VSV-G-pseudotyped HIV-2-Luc reporter viruses (2–5 ng of Gag p27 antigen). For DMOG treatment experiments, macrophages were pre-treated with 1 mM DMOG for 6 h prior to infection. After 48 h, cells were subjected to immunoblot analysis or luciferase assay to measure viral infectivity using the Bright-Glo Luciferase Assay System (Promega). For luciferase assays, cells were transferred to white-bottom 96-well plates prior to the addition of the luciferase substrate.

### Gene expression analysis

Total RNA was extracted using the RNeasy Mini Kit (Qiagen) and reverse transcribed to cDNA using ReverTra Ace (Toyobo) according to the manufacturers’ instructions. Gene expression was analyzed by qPCR using TB Green Premix Ex Taq II (Takara) and a CFX96 Real-Time PCR Detection System (Bio-Rad). For several experiments, PCR products were analyzed by electrophoresis on 1% agarose gels. The primer sequences used are listed in S1 Table.

### Proteomic analysis for prolyl hydroxylation identification

Synthetic Vpx peptides (3 µM each) were synthesized by Bio-Synthesis Inc. and incubated with 300 nM recombinant PHD3/EGLN3 protein (Active Motif, #81033) in PHD3 reaction buffer at 30°C for 2 h. Samples were then desalted using a C18-tip column [35] and analyzed with an Orbitrap Elite Hybrid Ion Trap Mass Spectrometer (Thermo) coupled to an Ultimate 3000 RSLC nano system (Thermo). Protein identification was performed using the MASCOT search engine version 2.5.1 (Matrix Science) and the Vpx amino acid sequence database downloaded from UniProtKB with the following parameters: peptide mass tolerance, ± 5 ppm; fragment mass tolerance, ± 0.5 Da; variable modifications: oxidation of methionine, hydroxylation of proline, and deamidation of asparagine and glutamine. We used a significance threshold of *P* < 0.05 for exporting the results of the MASCOT-assisted analysis. A peptide score ≥20 was adopted as the acceptance criteria for identification. Quantitative analysis of the peptides was performed using the Progenesis QI for proteomics software version 2.0 (Nonlinear Dynamics).

### Crosslinking mass spectrometry

Briefly, 1 μg HIV-2_Rod10_ Vpx (Aviva Systems, #OPCA03170) was incubated with 0.5–0.625 μg PHD3 (Active Motif, #81033) at 37°C for 1 h in PHD3 reaction buffer, followed by the addition of 1.83 μg VHL/EloBC complex (Abcam, #ab271497). The proteins were crosslinked using 5 mM DSBU (Thermo) at 25°C for 2 h, and the reaction was quenched with 100 mM NH_4_HCO_3_. The crosslinked samples were precipitated using trichloroacetic acid and washed twice with ice-cold acetone. The protein pellets were dissolved in 8 M urea buffer containing 75 mM NaCl and 50 mM Tris (pH 8.2). After reduction with dithiothreitol and alkylation with iodoacetamide, samples were diluted and digested overnight with trypsin at 37°C. The resulting peptides were desalted using Stage Tips.

LC–MS/MS analysis was performed on a Q Exactive HF mass spectrometer (Thermo) coupled to an Ultimate 3000 HPLC system (Thermo). Peptides were separated on a C18 nano-column (75 μm × 120 mm) and a 115 min gradient from 5%–33% acetonitrile in 0.1% formic acid. MS spectra were acquired in the 300–1,500 m/z range, with charge states 2+ to 8 + selected for MS/MS analysis.

Data analysis was conducted using SEQUEST HT in Proteome Discoverer 2.3 and XlinkX software. Searches were performed against the UniprotKB database with a 1% false discovery rate threshold. Crosslinked peptides were identified using XlinkX with a minimum score of 40. Carbamidomethyl (cysteine) was set as a fixed modification, while oxidation (methionine), hydroxylation (proline), DSBU Hydrolyzed (lysine [+214.095 Da]), and acetyl (protein N-terminus) were set as variable modifications.

### Computational modeling of protein structures

The structures of Vpx and PHD3 were predicted using AlphaFold3 [36]. Subsequently, protein–protein docking was conducted using ClusPro [37], which generated 58 distinct Vpx-PHD3 complex models. The optimal model was selected based on two criteria derived from the crosslinking MS data: (1) spatial proximity of PHD3 Lys48 to Vpx Lys84 and (2) close positioning of PHD3 catalytic residues (His135, Asp137, His196) to Vpx Pro41, the putative hydroxylation site. Structural figures were prepared using the PyMOL Molecular Graphics System, Version 2.5.3 (Schrödinger).

### Vpx sequence analysis

To analyze similarity, the sequence data of the Vpx gene was downloaded from GenBank (accessed on Oct 15, 2024). Strains with ambiguous nucleotides or incomplete sequences were excluded. After removing 100% identical sequences, 80 HIV-2 Vpx genes were retained for analysis. The Vpx amino acid sequences were aligned using Geneious Prime 2024.0.7 (https://www.geneious.com). The non-synonymous (dN) and synonymous (dS) substitution rates at each amino acid site were calculated using the Datamonkey web server [38]. Three complementary approaches were employed: Fixed Effects Likelihood (FEL) [39], Fast Unconstrained Bayesian AppRoximation (FUBAR) [40], and Single-Likelihood Ancestor Counting (SLAC) [39]. The significance level was set at *P* < 0.05 for FEL and SLAC analyses. For FUBAR, evidence of selective pressure was supported by a posterior probability > 0.9.

The nucleotide similarity among the aligned sequences of the HIV-2 Vpx gene was analyzed using SimPlot version 3.5.1 [41]. NCBI Reference Sequence (NC_001722) was used as the reference sequence based on its complete annotation and widespread use in previous studies. The similarity was examined using the Kimura 2-parameter method with a window size of 200 nt and a step size of 20 nt.

### Phylogenetic analysis

Multiple alignments for these sequences were performed using MAFFT version 7.520 [42]. Maximum likelihood phylogenetic analysis was performed using IQ-TREE version 2.2.2.6 with Model Finder, ultrafast bootstrap test parameters, and SH-like approximate likelihood ratio test [43]. The resultant trees were visualized using FigTree version 1.4 (http://tree.bio.ed.ac.uk/software/figtree/).

### Statistical analysis

All results are presented as mean ± standard deviation. Statistical comparisons between two groups were conducted using a two-tailed unpaired *t*-test in GraphPad Prism version 9 software. Statistical significance was set at *P* value < 0.05.

## Supporting information

S1 Fig Gene knockdown efficiency of siRNA used in this study. (A, B) HEK293 cells expressing indicated siRNA targeting CULs (A) and PHDs (B) were subjected to qRT-PCR for gene expression analysis. Statistical significance was determined using a two-tailed unpaired *t*-test. ***P < 0.001; **P < 0.01.
(PDF)

S2 Fig Mass spectrometric analysis of hydroxylated Vpx. (A) Mass spectra showing hydroxylation of Vpx Pro41. (B) Hydroxylation status of the Vpx C-terminal peptide. Vpx peptides were incubated with recombinant PHD3 and analyzed by mass spectrometry.
(PDF)

S3 Fig Characterization of Vpx P41A mutation. (A, B) Vpx P41A mutation does not affect Vpx binding to SAMHD1 or TASOR. Co-immunoprecipitation of HEK293 cells expressing FLAG-Vpx (WT or P41A) and HA-SAMHD1 (A) or HA-TASOR (B) was performed using an anti-HA antibody. (C) PHD3 supplementation enhances Vpx ubiquitination by VHL. In vitro ubiquitination assay was performed as in Fig 1H, with or without the addition of recombinant PHD3 and its cofactors (Fe^2+^, 2-oxoglutarate, and ascorbate). (D) PHD3 depletion attenuates VHL-mediated Vpx ubiquitination. HEK293 cells were transfected with control (Ctrl) or PHD3-specific siRNAs and co-expressing FLAG-Vpx and HA-VHL. Cell lysates were then immunoprecipitated using an anti-FLAG antibody and the immunoprecipitates were analyzed by immunoblotting with anti-ubiquitin (Ub) and anti-FLAG antibodies.
(PDF)

S4 Fig Phylogenetic analysis of HIV-2 and SIV Vpx proteins. Maximum likelihood tree of the Vpx amino acid sequences was constructed using IQ-TREE 2.2.2.6 with JTT + G4 model of substitution. Branches are colored in red where Pro41 is conserved. The phylogenetic tree was constructed with the following strains: HIV-2 (AB499693, AB731743, AB731744, FJ594493, KU179861, KX174311, KX174313, KY025538, KY025539, KY025543, MF595855, MF595858, MF595861, MF595862, MF595865, MF595866, MH681607, MH681608, MH681611, OR333514, OR543074, OR543082, OR543084), SIVsmm (AF077017, AF334679, JX648292, JX860407, JX860413, JX860414, JX860415, JX860430, JX860431, JX860432, JX860433), SIVmac (M33262), SIVmnd (AF328295, AF367411, AY159322), SIVdrl (AY159321, KM378563), SIVrcm (AF349680, AF382829, HM803689, HM803690).
(PDF)

S5 Fig Crosslinking mass spectrometry analysis. Histogram shows the crosslinked peptides identified between PHD3 (VKQLHCTGALRDGQLAGPR) and Vpx (KGCTCLGR). The lysine residues (K) in both peptides were found to be crosslinked.
(PDF)

S1 Table Sequences of primers used in this study. (PDF)

S1 Data All quantitative data presented in figures. (XLSX)
