# Supplementary figures and images for: PHD3-VHL axis controls HIV-2 infection through oxygen-dependent hydroxylation and degradation of Vpx
Source: PLoS Pathog. 2025 Jun 16;21(6):e1013241. doi: 10.1371/journal.ppat.1013241 (PMC12201638; doi:10.1371/journal.ppat.1013241)

**A**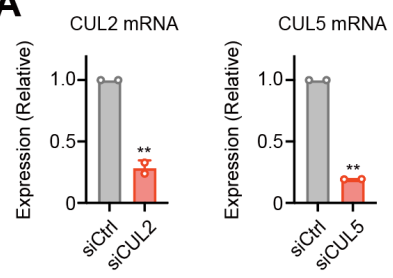**B**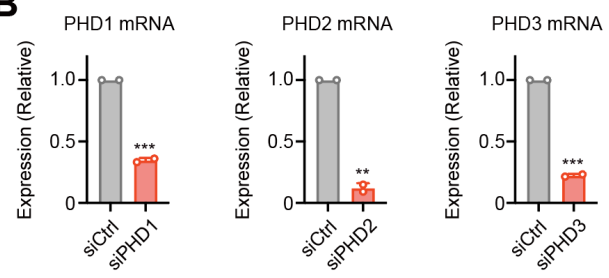

Supplement: S1 Fig — (A, B) HEK293 cells expressing indicated siRNA targeting CULs (A) and PHDs (B) were subjected to qRT-PCR for gene expression analysis. Statistical significance was determined using a two-tailed unpaired t-test. ***P < 0.001; **P < 0.01. (PDF) [file ppat.1013241.s001.pdf]

**A**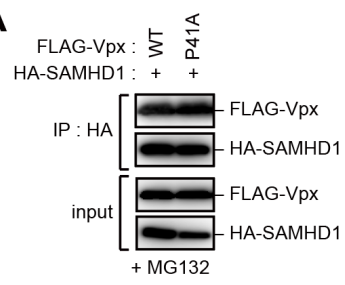**B**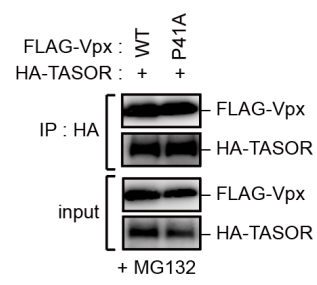**C**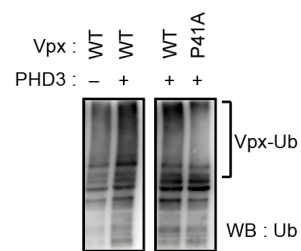**D**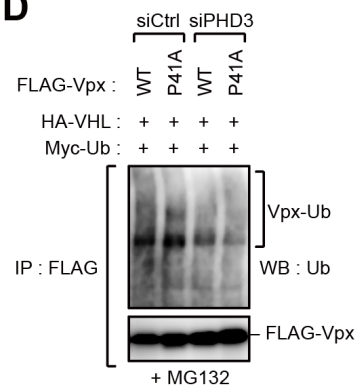

Supplement: S3 Fig — (A, B) Vpx P41A mutation does not affect Vpx binding to SAMHD1 or TASOR. Co-immunoprecipitation of HEK293 cells expressing FLAG-Vpx (WT or P41A) and HA-SAMHD1 (A) or HA-TASOR (B) was performed using an anti-HA antibody. (C) PHD3 supplementation enhances Vpx ubiquitination by VHL. In vitro ubiquitination assay was performed as in Fig 1H, with or without the addition of recombinant PHD3 and its cofactors (Fe2+, 2-oxoglutarate, and ascorbate). (D) PHD3 depletion attenuates VHL-mediated Vpx ubiquitination. HEK293 cells were transfected with control (Ctrl) or PHD3-specific siRNAs and co-expressing FLAG-Vpx and HA-VHL. Cell lysates were then immunoprecipitated using an anti-FLAG antibody and the immunoprecipitates were analyzed by immunoblotting with anti-ubiquitin (Ub) and anti-FLAG antibodies. (PDF) [file ppat.1013241.s003.pdf]

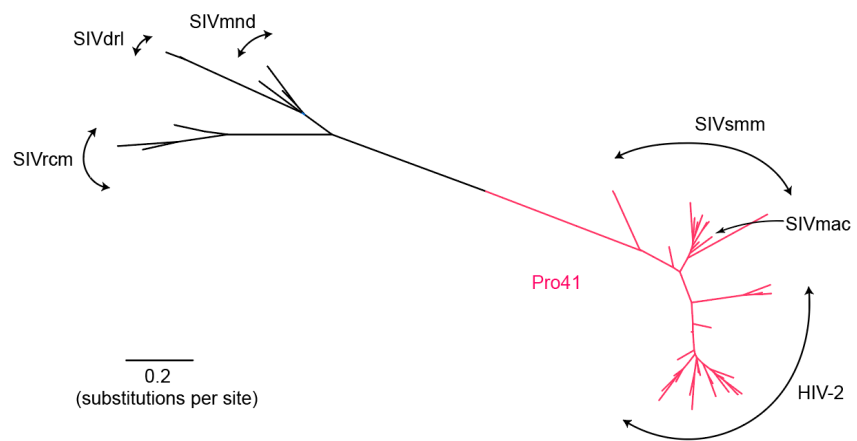

Supplement: S4 Fig — Maximum likelihood tree of the Vpx amino acid sequences was constructed using IQ-TREE 2.2.2.6 with JTT + G4 model of substitution. Branches are colored in red where Pro41 is conserved. The phylogenetic tree was constructed with the following strains: HIV-2 (AB499693, AB731743, AB731744, FJ594493, KU179861, KX174311, KX174313, KY025538, KY025539, KY025543, MF595855, MF595858, MF595861, MF595862, MF595865, MF595866, MH681607, MH681608, MH681611, OR333514, OR543074, OR543082, OR543084), SIVsmm (AF077017, AF334679, JX648292, JX860407, JX860413, JX860414, JX860415, JX860430, JX860431, JX860432, JX860433), SIVmac (M33262), SIVmnd (AF328295, AF367411, AY159322), SIVdrl (AY159321, KM378563), SIVrcm (AF349680, AF382829, HM803689, HM803690). (PDF) [file ppat.1013241.s004.pdf]

Vpx ( $\beta$ ) KGCTCLGR

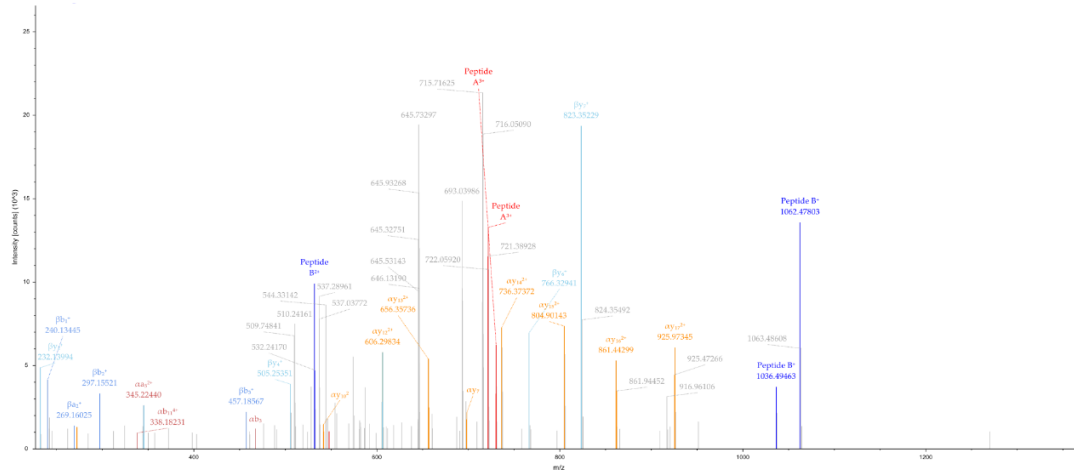

Supplement: S5 Fig — Histogram shows the crosslinked peptides identified between PHD3 (VKQLHCTGALRDGQLAGPR) and Vpx (KGCTCLGR). The lysine residues (K) in both peptides were found to be crosslinked. (PDF) [file ppat.1013241.s005.pdf]
