# Supplementary material for: PHD3-VHL axis controls HIV-2 infection through oxygen-dependent hydroxylation and degradation of Vpx
Source: PLoS Pathog. 2025 Jun 16;21(6):e1013241. doi: 10.1371/journal.ppat.1013241 (PMC12201638; doi:10.1371/journal.ppat.1013241)
Supplement: S2 Fig — (A) Mass spectra showing hydroxylation of Vpx Pro41. (B) Hydroxylation status of the Vpx C-terminal peptide. Vpx peptides were incubated with recombinant PHD3 and analyzed by mass spectrometry. (PDF) [file ppat.1013241.s002.pdf]

A

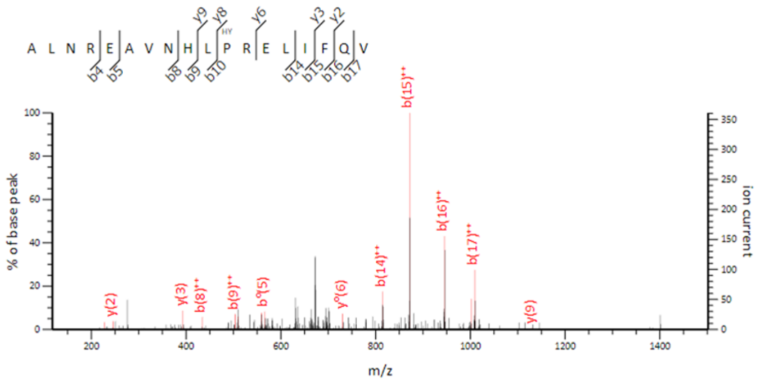
A L N R E A V N H L P R E L I F Q V
y9 y8 y6 y3 y2
HY
b4 b5 b8 b9 b10 b14 b15 b16 b17
b(15)**
b(16)**
b(17)**
b(14)**
y(2)
y(3)
b(8)**
b(9)**
b°(5)
y°(6)
y(9)
% of base peak
ion current
m/z

B

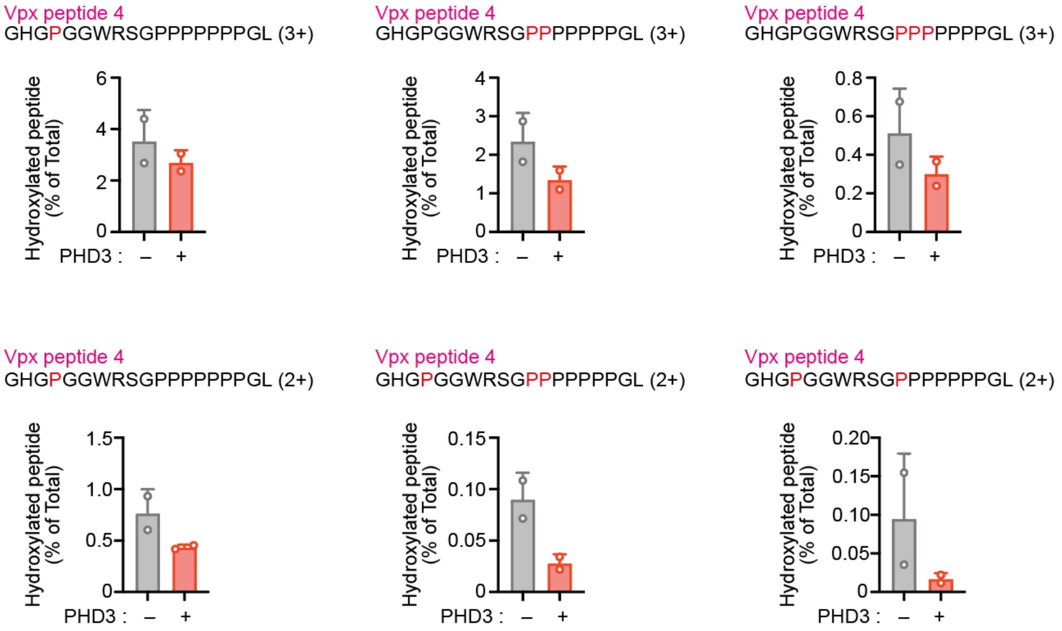
Vpx peptide 4
GHGPGGWRSGPPPPPPPPGL (3+)
Vpx peptide 4
GHGPGGWRSGPPPPPPPPGL (3+)
Vpx peptide 4
GHGPGGWRSGPPPPPPPPGL (3+)
Vpx peptide 4
GHGPGGWRSGPPPPPPPPGL (2+)
Vpx peptide 4
GHGPGGWRSGPPPPPPPPGL (2+)
Vpx peptide 4
GHGPGGWRSGPPPPPPPPGL (2+)
Hydroxylated peptide (% of Total)
PHD3 : – +
