# Supplementary material for: PHD3-VHL axis controls HIV-2 infection through oxygen-dependent hydroxylation and degradation of Vpx
Source: PLoS Pathog. 2025 Jun 16;21(6):e1013241. doi: 10.1371/journal.ppat.1013241 (PMC12201638; doi:10.1371/journal.ppat.1013241)
Supplement: S1 Table — (PDF) [file ppat.1013241.s006.pdf]

Target	Primer pair (5’ - 3’)
ACTB	GGACTTCGAGCAAGAGATGG
	AGCACTGTGTTGGCGTACAG
VHL	GACACACGATGGGCTTCTGGTT
	ACAACCTGGAGGCATCGCTCTT
CUL2	GTCTTACTCCGTGCTGTGTCCA
	CTGACTCCACAAATAGTGTTGGC
CUL5	CCTGATGCTGAACTTAGGAGGAC
	GGTTCACTGAGAAGAGGGTACC
PHD1	CTGTCTGGTATTTTGATGCCAAGG
	CGGCTGTGATACAGGTACTTGG
PHD2	TGAGCAGCATGGACGACCTGAT
	CGTACATAACCCGTTCCATTGCC
PHD3	GAACAGGTTATGTTCGCCACGTG
	CCCTCTGGAAATATCCGCAGGA
